# An Interest Stabilisation Mechanism to Unburden the ECB

**DOI:** 10.1007/s10272-021-0998-1

**Published:** 2021-10-05

**Authors:** Ivo Arnold

**Affiliations:** Erasmus School of Economics, Burgemeester Oudlaan 50, 3062 PA Rotterdam, Netherlands

## Abstract

Following the twin crises of sovereign debt and COVID-19, the ECB risks being stuck in a situation of fiscal dominance, in which monetary policy is subordinated to the needs of finance ministers. A strong post-COVID-19 recovery may increase inflationary pressures, requiring a shift towards a less accommodative monetary policy stance. A tightening of monetary policy may, however, lead to a widening of interest rate spreads and new bond market tensions in the euro area. This article argues that the credibility of the ECB is undermined if it is perceived as aiming to close interest spreads. Interest spreads between euro countries arising from fiscal concerns should be a matter of fiscal policy, not monetary policy. The establishment of an interest stabilisation mechanism would allow the ECB to restore monetary dominance and to focus on maintaining price stability.

Is Europe on the eve of a new roaring twenties period, as consumers start to spend the savings they hoarded during the pandemic? No one knows for sure, but central bankers should be prepared for such a post-COVID-19 scenario. After all, it is their job to take away the punch bowl just as the party gets going, to prevent that a post-pandemic party culminates in an inflation hangover. The European Central Bank (ECB) must thus stand ready to wind down its asset purchase programmes and return to a more neutral monetary policy stance with positive interest rates. For inflation expectations to remain anchored at low levels, it is important that firms, consumers and investors in financial markets have confidence in the ECB's ability to fight inflation when needed. That confidence risks being undermined if the ECB continues to use monetary policy to narrow the interest rate spreads between the more and less creditworthy euro countries and thus supports the weaker brethren in the union, as it has done since the start of the crisis. A tightening of monetary policy could lead to a widening of interest rate spreads and new tensions in the euro area. This article argues that it is better to solve this problem with fiscal policy instead of monetary policy, for example, by introducing a fiscal mechanism to dampen interest rate spreads.

**Ivo Arnold**, Erasmus University Rotterdam; and Nyenrode Business Universiteit, Breukelen, Netherlands.

## Fiscal dominance in times of crisis

In recent years, the ECB's asset purchase programmes have depressed long-term bond market yields and kept interest rate spreads in the euro area countries low. In this way, the ECB has enabled national governments to focus all their attention on tackling the COVID-19 crisis without having to worry about the bond market vigilantes. Falling tax revenues and rising government spending have sharply increased the budget deficits of European governments in the past year. The policy of the ECB has ensured that these deficits could be financed at a low interest rate. In the heat of the crisis, this was arguably the right policy: The central bank had to remove any doubts in the financial markets about the solvency of the government. For example, during World War II, the US national debt rose from 40% to more than 100% of GDP to fund the war effort. This increase went about smoothly because the Federal Reserve kept interest rates very low by buying up treasury bonds.

A situation in which monetary policy is subordinated to meet the needs of finance ministers is called fiscal dominance. Northern European countries that value price stability and fiscal discipline, such as the Netherlands and Germany, generally feel uneasy about such a state of affairs. It is not what these countries have signed up for in the Maastricht Treaty, which established the ECB as an independent central bank whose main objective is price stability. That is the reason why the ECB is doing its utmost to explain to the public that the asset purchase programmes are still within its mandate and that we have not arrived in a state of fiscal dominance. For example, ECB board member Schnabel (2020) pointed out that inflation expectations in the euro area have remained low during the crisis and that the asset purchase programmes are primarily aimed at stabilising the financial markets. Market stabilisation is needed to prevent "bad equilibria", in which unfounded panic in the bond markets pushes the interest rates of weak euro countries to levels so high that this raises doubts about the sustainability of their public finances. But what the ECB sees as stabilising the financial markets can also be interpreted as an interest rate subsidy to euro countries with a low creditworthiness. In the latter interpretation, it would be more appropriate to tackle this problem with fiscal policy than with monetary policy.

### Time for the ECB to choose

During the latest crisis, the ECB's policy of keeping down governments' funding costs was compatible with its mandate of price stability. Inflation was too low rather than too high. Southern Europe even saw some signs of deflation. But as vaccination rates rise and inflation expectations increase, the ECB must make a choice. Will the ECB restore monetary dominance and shift its ultra-accommodative monetary policy to a more neutral stance? Or will the ECB continue to keep the borrowing costs of weak euro countries under control, as is now the perception among investors and credit rating agencies. In the current institutional environment, with limited fiscal risk sharing, it is not obvious at all that the ECB will succeed in restoring monetary dominance. In March 2020, ECB President Christine Lagarde's attempt to emphasise the monetary dominance of ECB policy backfired. Her unfortunate remark that the ECB is not there to close spreads fuelled the turmoil in the bond markets and elicited angry reactions from Southern European politicians. The Pandemic Emergency Purchase Programme, especially designed to enable the ECB to deviate from the capital key and to purchase more debt from weak euro countries, then did what it had to do: narrow the interest rate spreads. For the ECB, the challenge therefore is to get out of a situation where monetary policy is being held hostage by fears of widening spreads.

### Avoiding the debt trap

Winding down the asset purchase programmes will have financial implications. Without support from the ECB, interest spreads in the euro area bond markets may widen again. We do not know by how much, but the euro area should be prepared for a strong reaction in the financial markets and for the possible consequences for the European economy. The coronavirus crisis has hit tourism-dependent Southern European countries hard and has led to a sharp rise in their public debt, making them more vulnerable to unfavourable debt dynamics. Given the low growth of their nominal GDP, these countries are in danger of falling into a debt trap, where the combination of deficits, high interest rates and low economic growth projects an unsustainable future path for the debt-to-GDP ratio (see e.g. Padoan et al., 2012). Financial market concerns about debt sustainability will then further increase bond yields. Lacking ECB support, weak euro area member countries may end up in this vicious circle.

Some will argue that responsibility for fiscal policy lies at the national level and that distressed euro area countries should quickly put their public finances in order. But such an every-man-for-himself strategy has many drawbacks, even for strong euro area countries. As we have seen in the period following the global financial crisis, it can lead to countries adopting too harsh austerity policies, with negative consequences for economic growth and popular support for European integration (Blyth, 2013; De Grauwe and Ji, 2013).

Countries can also try to meet their financing needs by issuing short-term debt to avoid having to issue long-term bonds at high yields and in the hope that bond yields will fall again soon. The drawback of this approach is that it will turn public debt management into a potential source of fiscal instability. There are indications that this has happened during the euro crisis. Using data from ten countries in the euro area, Arnold (2021) finds that sovereign risk is a significant variable in debt reaction functions: Higher sovereign default spreads are positively related to short-term debt issuance.

A further finding in the literature is that in the past decade, the ECB's refinancing operations have enabled banks located in distressed countries to invest in domestic public debt and thereby stimulate the demand for long-term debt issues (Acharya and Steffen, 2015; Arnold and Soederhuizen, 2018). When ECB support is withdrawn and sovereign spreads on long-term bonds start to increase, debt issuance may tilt back to shorter maturities. The disadvantage of such a short-term funding strategy is that weak euro countries will have to refinance their debt more often, which increases their vulnerability to a funding crisis and makes the euro area financially less stable.

Another argument against the every-man-for-himself strategy is that fiscal instability can easily spill over into the European financial system. A large literature documents the interconnectedness between banks and sovereigns in the euro area and the risk of the doom loop between banking and sovereign risk (see e.g. Alter and Schuler, 2012; Acharya et al., 2014; Alter and Beyer, 2014; Brunnermeier et al., 2016). Policymakers also regard this doom loop as a financial stability concern (European Systemic Risk Board, 2015; Andritzky et al., 2016; Van Riet, 2017). Any damage to the European banking sector following a reignition of fiscal tensions will compromise the uniform transmission of monetary policy in the euro area.

Finally, the flaring up of tensions in the European bond markets could lead investors to seek a safe haven in the most creditworthy countries, as a result of which their interest rates will remain too low. High interest rate spreads within the currency union thus lead to a situation of financial fragmentation, in which monetary policy does not have the same effect on interest rates and the economy everywhere in the currency union.

In short, widening interest spreads between euro countries hinder the ECB in its monetary policy. But if the ECB tries to close spreads, it risks stepping outside its mandate and feeds the unease about the legitimacy of the asset purchase programmes. Although the recent legal challenge of the German Constitutional Court has not forced the ECB to change tack, future challenges may be more successful. Out of caution, it would thus make sense to search for another way to manage spreads, so that the ECB can focus on its primary objective of price stability.

## Emergency funding

Since the euro crisis, hundreds of billions of euros in European emergency and support funding have been made available to distressed countries. The European Stability Mechanism (ESM) provides emergency loans to euro countries that run into financial difficulties. However, since its inception, concerns have been raised about its limited size (see e.g. Gros and Mayer, 2012). After the COVID-19 outbreak, the Recovery and Resilience Facility (RRF), which consists of a combination of cheap loans and grants, has been added to the ESM funding. While these funds may contribute to the mitigation of fiscal crises, they also have drawbacks. Countries do not like to draw from the ESM either because they do not want to be stigmatised or because they do not want to commit to the associated economic reforms. The RRF works on the basis of reform plans and project proposals, making it a slow, cumbersome and bureaucratic instrument.

The ESM and the RRF have also failed to eliminate the need for ECB intervention in the bond markets. All support programmes, including the public sector purchase programmes and the (unused) outright monetary transactions of the ECB, have the common feature that European public funding replaces private market funding of weak euro countries. This could imply that, in the long run, most Southern European government debt will be held by European institutions. This is an undesirable outcome. A different approach would focus not on loans, but on interest costs.

## An interest stabilisation mechanism to unburden the ECB

In addition to providing emergency loans, the institution of the ESM can be used to set up a mechanism to dampen or smooth interest rate spreads within the euro area. Proposals for such an interest stabilisation mechanism (ISM) have been made previously in Arnold (2012) and Heinemann (2012). The basic idea is that in the event of bond market unrest, when capital flees from Southern to Northern Europe and spreads widen, the rising interest costs of distressed countries are subsidised using the interest rate windfalls of the AAA countries. Ideally, such a mechanism is activated early and automatically, for example, when the interest rate spreads exceed a threshold value. The precise value at which the mechanism kicks in could be derived from an analysis of the debt sustainability of a euro country, making use of the condition that for debt sustainability the difference between interest costs and nominal GDP growth should remain below the primary surplus (Gros, 2012). Early and automatic activation is important for several reasons. It prevents the crisis response from lagging behind the curve, as has been the case in the past. It also prevents countries from resorting to short-term debt as long-term yields increase. Finally, it reduces bond market volatility and limits speculators' opportunities to engage in convergence plays.

In its most extreme form, all interest costs in the euro area could be pooled and evened out in time leading to an equalisation of the ratios of interest costs to total debt across countries. A less radical implementation would focus on distressed countries only and subsidise unsustainable interest rate payments on newly issued long-term debt. This could achieve the objective of breaking the vicious circle of the debt trap, in which rising interest costs cast doubt on the sustainability of government debt and further drive up market rates. An ISM breaks the pass-through of rising market interest rates to the interest cost of individual distressed countries and thus short-circuits the debt trap. This feature will in itself have a stabilising effect on the financial markets. Weak euro countries thus retain access to the capital market and will not be tempted to resort to short-term funding. They are given time for economic reforms and can avoid costly short-term austerity measures.

Compared to the existing emergency and support funds, an ISM has several advantages for the strong euro countries. First, an ISM does not require hundreds of billions of euros to be put on the table, as interest subsidies can be drip-fed to distressed countries when they issue long-term debt in challenging market conditions. Heinemann (2012) and Dixon (2012) have calculated that such a mechanism is feasible. A second advantage is that sovereign debt is not mutualised, as in the case of eurobonds, and that the individual countries remain liable for their own sovereign bonds. Notwithstanding the agreements on the ESM and RRF, it remains difficult for Northern European politicians to explain to voters why they should guarantee Southern European debt. It may be easier to explain how an ISM contributes to financial stability in the euro area by recycling the windfalls from the safe-haven effect to Southern European countries. Third, as with the ESM's emergency loans, conditions can be attached to the ISM, such as an economic reform programme. The interest rate subsidies can then be made conditional on sufficient progress in economic reforms and stopped when countries balk at implementing reforms. Fourth, the introduction of an ISM could be tied to reform of the regulatory treatment of sovereign exposures of banks in the euro area. The lack of risk weightings on sovereign bonds and their exemption from the large exposure directive enables governments to put pressure on their domestic banks to invest in domestic sovereign bonds. While this reduces bond yields, it also has the negative effect of strengthening the sovereign-bank nexus and increasing the transmission of sovereign risk to the banking system. Reform of the regulatory treatment of sovereign exposures has, however, stalled. In its roadmap for the completion of the monetary union, the European Commission (2017) showed little urgency for reform in this area. Obviously, weak euro countries are concerned that stricter rules on sovereign exposures will force them to fund more public debt in the financial markets at higher interest rates. Applying non-zero risk weightings and diversification rules to sovereign exposures will thus require a difficult adjustment process, but an ISM could provide the safety net that these governments need to take the necessary steps in banking regulation.

The main advantage of an ISM is, however, that it will protect the credibility of the ECB. It allows the ECB to remain within its mandate, to restore monetary dominance and to focus on maintaining price stability. An ISM makes it clear that the reduction of interest spreads between euro area member countries arising from fiscal concerns is a matter of fiscal policy, not monetary policy. With an ISM in place, Christine Lagarde can rightly and credibly say, that we are not here to close spreads.
